# Schizophrenia and hetero-aggressiveness: management and aggravating factors

**DOI:** 10.1192/j.eurpsy.2023.2290

**Published:** 2023-07-19

**Authors:** M. Chtibi, H. Berrada, I. Hanine, S. Belbachir, A. Ouanass

**Affiliations:** Arrazi University Psychiatric Hospital of Salé, Faculty of Medicine and Pharmacy of Rabat, Rabat, Morocco

## Abstract

**Introduction:**

Schizophrenia is a severe mental illness but especially important in terms of its impact on the subject. The stigmatization of these patients is major, leading to a significant decrease in their quality of life. This is partly due to the media coverage of the rare cases of hetero-aggression.

The aggressiveness of schizophrenic subjects remains poorly known and little studied.

**Objectives:**

The objectives of our study are to determine whether the prescription of second-generation antipsychotics is associated with lower levels of aggression than the prescription of first-generation antipsychotics and to identify aggravating factors.

**Methods:**

Materials and methods: We used an anonymous questionnaire based on, in addition to individual status and conditions, a self-administered questionnaire to assess the degree of aggression (the Buss and Perry Aggression Questionnaire (BPAQ)).

**Results:**

Our study demonstrated superiority of second-generation antipsychotics in preventing aggression in subjects with schizophrenia, as well as an association between increased aggression and low insight, low compliance and low social support. In addition, younger age, male gender, and lower education were associated with increased aggression.

**Conclusions:**

The prevention of aggression would then begin with the management of psychotic symptoms and comorbid disorders, as well as work on the compliance and insight of these patients. However, the aggressive dimension persists in some of them.

**Disclosure of Interest:**

None Declared

